# The complete mitochondrial genome of the Endangered European brown frog *Rana pyrenaica* through RNAseq

**DOI:** 10.1080/23802359.2016.1174087

**Published:** 2016-07-05

**Authors:** Marcos Peso-Fernández, Raquel Ponti de la Iglesia, Guillermo Ponz Segrelles, Rubén González Martínez, Angel Arcones Segovia, David R. Vieites

**Affiliations:** a Museo Nacional De Ciencias Naturales – CSIC, Madrid, Spain;; b BioCoRe, Calle Zurbarán 16, Collado Mediano, Madrid, Spain;; c CIBIO-InBIO, Universidade Do Porto, Campus Agrário De Vairão, Vairão, Portugal

**Keywords:** Amphibia, mitogenome, Pyrenees, *Rana pyrenaica*, RNAseq

## Abstract

We sequenced the complete mitogenome of the Pyrenean frog *Rana pyrenaica*, which was determined from an Illumina Hi-seq RNAseq run (Illumina Inc., San Diego, CA). The genome is 17,213 bp in size, including 13 protein-coding genes, 21 transfer RNAs, two ribosomal RNAs and a control region. It shows the typical gene order of previously available frog mitogenomes, although it lacks the *tRNA^Phe^*. This is the first complete mitogenome described for a Western Palearctic brown frog species.

The Pyrenean frog (*Rana pyrenaica*) is an Endangered narrowly distributed endemism (Sillero et al. 2014). Despite that its sister species *Rana temporaria* shows a considerable degree of mtDNA genetic variation across its range, including several divergent lineages in the Pyrenees (Vences et al. 2013); a preliminary mtDNA study across the range of *R. pyrenaica* showed a single mutation difference using three mitochondrial genes (Carranza & Arribas 2008). This potential lack of genetic variation has important conservation implications for this Endangered species.

RNAseq is becoming a common approach for gathering transcriptome data using Next-Generation Sequencing, being complete mitogenomes a potential output. We explored the use of RNAseq to describe the complete mitogenome of *R. pyrenaica*, which will benefit future phylogeographic, population genetic and conservation studies.

An adult of *R. pyrenaica* (Museo Nacional de Ciencias Naturales, Madrid, collection number MNCN 46671) was collected in Uztárroz (42°35′38″N, 0°59′27″W), NE Spain. We extracted RNA from several tissues, which were quantified with Qubit HS and normalized. A RNAseq library was prepared using the NEBNext Ultra RNA kit for Illumina (Illumina Inc., San Diego, CA). Quantification and size estimation were performed on a Bioanalyzer 2100 High Sensitivity DNA chip, and sequenced on 1/2 lane on a Illumina HiSeq (2 × 100 bp pair-end reads). After quality control and trimming with Trimmomatic (v 0.32.2) (Bolger et al. 2014), assembly was done with Trinity (v 2.0.6) (Haas et al. 2013). Trinity recovered the mitogenome except part of the control region; hence we used this assembly as input for MITObim (Hahn et al. 2013) to complete the reconstruction. Genome annotation was done through nucleotide sequence alignments with other ranids. The genome is deposited in GenBank (KU720300).

The complete mitogenome of *R. pyrenaica* is 17,213 bp in length, including 13 protein-coding genes, two rRNAs, 21 tRNAs and a control region. Gene order, lengths and codon compositions are shown in Table 1. The overall base composition of the heavy strand is 27.7% for A, 28.3% for T, 14.9% for G and 29.1% for C, with an A + T bias of 59.9%, similar to other ranid species (Hofman et al. 2014, Li et al. 2014a, 2014b, Ni et al. 2015). The genome shows a similar gene organization as other ranids (Kurabayashi et al. 2010; Xia et al. 2014), but it is the only anuran known so far lacking the *tRNA^Phe^*. A maximum-likelihood phylogenetic analysis of ranid frogs based on the available complete mitogenomes (Figure 1) recovers the European *R. pyrenaica* as the sister taxon to the clade of Asian brown frogs.

**Figure 1.**
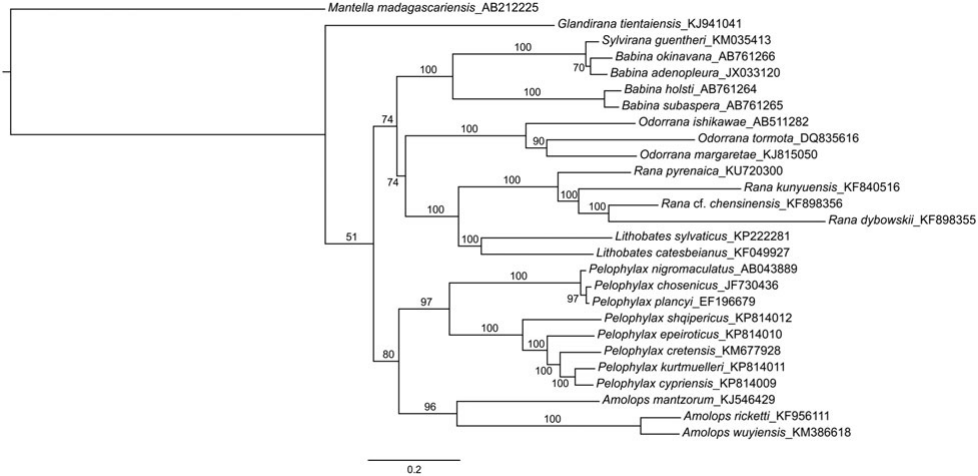
Phylogenetic reconstruction of the relationships between ranid frogs, based on available complete mitochondrial genomes except control regions. Maximum-likelihood analyses using a partitioned dataset by codon and gene were performed in RaxML, running for 1000 generations. ML support values are provided above branches. Genbank accession numbers are provided after the species names.

**Table 1.** Location of features in the mtDNA of *R. pyrenaica*.

Gene/region	Start position	Stop position	Length (bp)	Spacer (+) overlap (−)	Start codon	Stop codon	Strand
tRNA^Leu^	1	72	72	2			H
tRNA^Thr^	75	144	70	0			H
tRNA^Pro^	145	176	32	3			H
*12S rRNA*	180	1109	930	0			H
tRNA^Val^	1109	1177	69	−1			H
*16S rRNA*	1178	2758	1581	1			H
tRNA^Leu^ (UUR)	2760	2832	73	0			H
*NAD1*	2833	3793	961	0	ATT	T––	H
tRNA^Ile^	3794	3863	71	0			H
tRNA^Gln^	3864	3934	71	−1			H
tRNA^Met^	3934	4002	69	−1			H
*NAD2*	4003	5035	1033	0	ATT	T––	H
tRNA^Trp^	5036	5105	70	0			H
tRNA^Ala^	5106	5175	70	0			L
tRNA^Asn^	5176	5248	73	0			L
*OL*	5249	5278	30	0			L
tRNA^Cys^	5276	5340	65	−3			L
tRNA^Tyr^	5341	5407	67	3			L
*COI*	5411	6961	1551	0	ATA	AGG	H
tRNA^Ser^ (UCN)	6953	7023	71	−9			L
tRNA^Asp^	7025	7093	69	0			H
*COII*	7094	7781	688	0	ATG	T––	H
tRNA^Lys^	7782	7850	69	1			H
*ATP8*	7852	8013	162	0	ATG	TAA	H
*ATP6*	8007	8688	682	−7	ATG	T––	H
*COIII*	8689	9472	784	0	ATG	T––	H
tRNA^Gly^	9473	9540	68	0			H
*ND3*	9541	9880	340	0	ATG	T––	H
tRNA^Arg^	9881	9949	69	0			H
*ND4L*	9950	10,234	285	0	ATG	TAA	H
*ND4*	10,228	11,587	1360	−7	ATG	T––	H
tRNA^His^	11,588	11,655	68	0			H
tRNA^Ser^ (AGY)	11,656	11,722	67	30			H
*ND5*	11,753	13,544	1792	105	ATG	T––	H
*ND6*	13,650	14,150	501	0	ATG	AGA	L
tRNA^Glu^	14,151	14,218	68	3			L
*Cyt b*	14,222	15,364	1143	0	ATG	TAA^a^	H
*Control region*	15,365	17,211	1846	0			H

a Stop codon completed with the addition of an A.

RNAseq has been proven to be a very fast and useful approach to gather complete mitogenomes. Although using common assembly tools like Trinity was not enough to gather the full genome, the combination with MITOBim has performed well to fill the assembly gaps.
